# 1-(2,3,5,6-Tetra­methyl­benz­yloxy)-1*H*-benzotriazole

**DOI:** 10.1107/S1600536809010794

**Published:** 2009-03-28

**Authors:** B. Ravindran Durai Nayagam, Samuel Robinson Jebas, J. P Edward Rajkumar, Dieter Schollmeyer

**Affiliations:** a Department of Chemistry, Popes College, Sawyerpuram 628 251, Tamilnadu, India; b Department of Physics, Karunya University, Karunya Nagar, Coimbatore 641 114, India; c Department of Physics, Popes College, Sawyerpuram 628 251, Tamilnadu, India; d Institut für Organische Chemie, Universität Mainz, Duesbergweg 10-14, 55099 Mainz, Germany

## Abstract

In the title compound, C_17_H_19_N_3_O, the benzotriazole ring is essentially planar, with a maximum deviation of 0.0069 (15) Å. The mean plane of the benzotriazole ring forms a dihedral angle of 13.16 (4)° with the mean plane of the benzene ring. The crystal packing is stabilized by π–π stacking inter­actions, with a centroid–centroid distance of 3.8077 (12) Å, together with weak C—H⋯π inter­actions. Mol­ecules are stacked along the *a* axis.

## Related literature

For bond-length data, see: Allen *et al.* (1987 ▶). For the biological activity of *N*-oxide and benzotriazole derivatives, see: Katarzyna *et al.* (2005 ▶); Sarala *et al.* (2007 ▶). For applications of benzotriazole, see: Kopec *et al.* (2008 ▶); Krawczyk & Gdaniec (2005 ▶); Smith *et al.* (2001 ▶); Sha *et al.* (1996 ▶). For 1-hydroxy­benzotriazole, see: Anderson *et al.* (1963 ▶); Bosch *et al.* (1983 ▶).
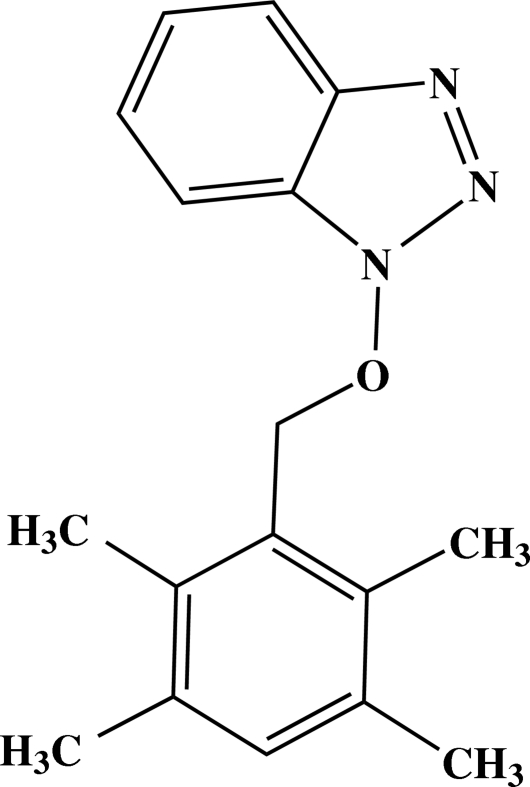


## Experimental

### 

#### Crystal data

C_17_H_19_N_3_O
*M* *_r_* = 281.35
Monoclinic, 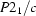

*a* = 4.9737 (8) Å
*b* = 26.3838 (18) Å
*c* = 11.490 (2) Å
β = 105.977 (7)°
*V* = 1449.5 (4) Å^3^
*Z* = 4
Cu *K*α radiation
μ = 0.65 mm^−1^
*T* = 193 K
0.51 × 0.51 × 0.45 mm

#### Data collection

Enraf–Nonius CAD-4 diffractometer
Absorption correction: ψ scan (*CORINC*; Draeger & Gattow, 1971 ▶) *T*_min_ = 0.731, *T*_max_ = 0.759
2861 measured reflections
2729 independent reflections
2578 reflections with *I* > 2σ(*I*)
*R*_int_ = 0.050
3 standard reflections frequency: 60 min intensity decay: 1%

#### Refinement

*R*[*F* ^2^ > 2σ(*F* ^2^)] = 0.047
*wR*(*F* ^2^) = 0.135
*S* = 1.08
2729 reflections
195 parameters
H-atom parameters constrained
Δρ_max_ = 0.27 e Å^−3^
Δρ_min_ = −0.23 e Å^−3^

### 

Data collection: *CAD-4 EXPRESS* (Enraf–Nonius, 1994 ▶); cell refinement: *CAD-4 EXPRESS*; data reduction: *CORINC* (Draeger & Gattow, 1971 ▶); program(s) used to solve structure: *SHELXS97* (Sheldrick, 2008 ▶); program(s) used to refine structure: *SHELXL97* (Sheldrick, 2008 ▶); molecular graphics: *SHELXTL* (Sheldrick, 2008 ▶); software used to prepare material for publication: *SHELXTL* and *PLATON* (Spek, 2009 ▶).

## Supplementary Material

Crystal structure: contains datablocks global, I. DOI: 10.1107/S1600536809010794/at2749sup1.cif

Structure factors: contains datablocks I. DOI: 10.1107/S1600536809010794/at2749Isup2.hkl

Additional supplementary materials:  crystallographic information; 3D view; checkCIF report

## Figures and Tables

**Table 1:** Hydrogen-bond geometry (Å, °)

*D*—H⋯*A*	*D*—H	H⋯*A*	*D*⋯*A*	*D*—H⋯*A*
C18—H18*C*⋯*Cg*3^i^	0.98	2.85	3.6701 (18)	141
C20—H20*B*⋯*Cg*3^ii^	0.98	2.80	3.682 (2)	150

Symmetry codes: (i) 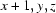; (ii) 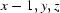. *Cg*3 is the centroid of the C12–C17 ring.

**Fig. 1.**
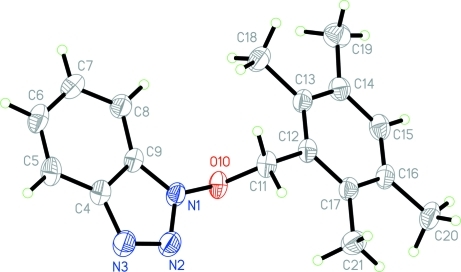
The molecular structure of the title compound, showing 50% probability displacement ellipsoids and the atom numbering scheme.

**Fig. 2.**
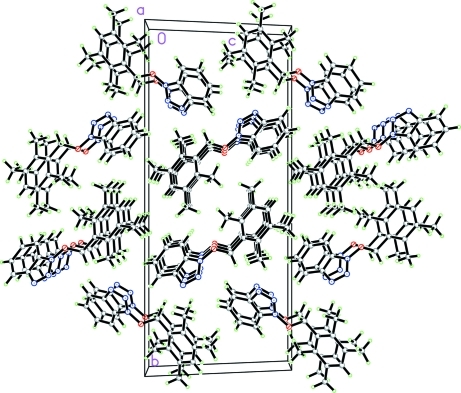
The crystal packing of the title compound, viewed down the a axis. Molecules are stacked along the a axis.

## supplementary crystallographic information

### Comment

Benzotriazole derivatives show biological activities such as anti-inflammatory, diuretic, antiviral and antihypertensive agents (Katarzyna *et al.*, 2005; Sarala *et al.*, 2007). It is used as a corrosion inhibitor, antifreeze agent, ultraviolet light stabilizer for plastics and as an antifoggant in photography (Krawczyk & Gdaniec, 2005; Smith *et al.*, 2001). *N*-aryloxy derivatives of benzotriazoles have antimycobacterial activity (Kopec *et al.*, 2008). Benzotriazole possessing three vicinal N atoms, is used as an antifouling and antiwear reagent (Sha *et al.*, 1996). 1-Hydroxybenzotriazole is widely being used as a reagent for peptide synthesis (Anderson *et al.*, 1963). The crystal structure of benzotriazole 1-oxide has been reported (Bosch *et al.*, 1983). Due to the above mentioned applications of benzotriazole we have synthesized and report here the crystal structure of the title compound (I).

The asymmetric unit of (I) comprises of one molecule of the title compound (Fig 1). The bond lengths and angles are found to have normal values (Allen *et al.*, 1987). The benzotriazole ring is essentially planar with the maximum deviation from planarity being 0.0069 (15) Å for atom N2. The mean plane of the benzotriazole ring (N1—N3/C4—C9) forms a dihedral angle of 13.16 (4)° with the mean plane of the phenyl ring (C12—C17).

The crystal packing is stabilized by π—π stacking interactions [*Cg*1—*Cg*2^i^= of 3.8077 (12) Å; *Cg*1: (N1—N3/C4—C9); *Cg*2:(C4—C9): symmetry code:(i) -1+x, y, z] together with weak C—H···π interactions. Molecules are stacked along the *a* axis (Fig.2).

### Experimental

A mixture of 1-(bromomethyl) 2,3,5,6 tetramethyl benzene (0.227 g, 1 mmol) and sSodium salt of 1-hydroxybenzotriazole (0.1571 mmol) in ethanol (10 ml) was heated at 333 K with stirring for 30 min. The compound formed was filtered off, and dried. The compound was dissolved in ethanol and on slow evaporation crystals suitable for *x*-ray diffraction are formed.

### Refinement

All the H atoms were positioned geometrically (C—H = 0.95 Å (aromatic); C—H = 0.98 (methyl) or C—H = 0.99 Å (methylene) and refined using a riding model with, *U*_iso_(H) = 1.2U_equ_(C_aromatic, methylene_) and 1.5U_equ_(C_methyl_). A rotating group model was used for the methyl groups.

### Crystal data

**Table:**

C_17_H_19_N_3_O	*F*(000) = 600
*M**_r_* = 281.35	*D*_x_ = 1.289 Mg m^−^^3^
Monoclinic, *P*2_1_/*c*	Cu *K*α radiation, λ = 1.54178 Å
Hall symbol: -P 2ybc	Cell parameters from 25 reflections
*a* = 4.9737 (8) Å	θ = 65–70°
*b* = 26.3838 (18) Å	µ = 0.65 mm^−^^1^
*c* = 11.490 (2) Å	*T* = 193 K
β = 105.977 (7)°	Block, colourless
*V* = 1449.5 (4) Å^3^	0.51 × 0.51 × 0.45 mm
*Z* = 4	

### Data collection

**Table:**

Enraf–Nonius CAD-4 diffractometer	2578 reflections with *I* > 2σ(*I*)
Radiation source: rotating anode	*R*_int_ = 0.050
graphite	θ_max_ = 69.9°, θ_min_ = 3.4°
ω/2θ scans	*h* = −5→6
Absorption correction: ψ scan (CORINC; Draeger & Gattow, 1971)	*k* = 0→32
*T*_min_ = 0.731, *T*_max_ = 0.759	*l* = −13→0
2861 measured reflections	3 standard reflections every 60 min
2729 independent reflections	intensity decay: 1%

### Refinement

**Table:**

Refinement on *F*^2^	Secondary atom site location: difference Fourier map
Least-squares matrix: full	Hydrogen site location: inferred from neighbouring sites
*R*[*F*^2^ > 2σ(*F*^2^)] = 0.047	H-atom parameters constrained
*wR*(*F*^2^) = 0.135	*w* = 1/[σ^2^(*F*_o_^2^) + (0.0664*P*)^2^ + 0.687*P*] where *P* = (*F*_o_^2^ + 2*F*_c_^2^)/3
*S* = 1.08	(Δ/σ)_max_ < 0.002
2729 reflections	Δρ_max_ = 0.27 e Å^−^^3^
195 parameters	Δρ_min_ = −0.23 e Å^−^^3^
0 restraints	Extinction correction: *SHELXL97* (Sheldrick, 2008), Fc^*^=kFc[1+0.001xFc^2^λ^3^/sin(2θ)]^-1/4^
Primary atom site location: structure-invariant direct methods	Extinction coefficient: 0.0042 (6)

### Special details

**Table:**

Geometry. All e.s.d.'s (except the e.s.d. in the dihedral angle between two l.s. planes) are estimated using the full covariance matrix. The cell e.s.d.'s are taken into account individually in the estimation of e.s.d.'s in distances, angles and torsion angles; correlations between e.s.d.'s in cell parameters are only used when they are defined by crystal symmetry. An approximate (isotropic) treatment of cell e.s.d.'s is used for estimating e.s.d.'s involving l.s. planes.
Refinement. Refinement of *F*^2^ against ALL reflections. The weighted *R*-factor *wR* and goodness of fit *S* are based on *F*^2^, conventional *R*-factors *R* are based on *F*, with *F* set to zero for negative *F*^2^. The threshold expression of *F*^2^ > σ(*F*^2^) is used only for calculating *R*-factors(gt) *etc*. and is not relevant to the choice of reflections for refinement. *R*-factors based on *F*^2^ are statistically about twice as large as those based on *F*, and *R*- factors based on ALL data will be even larger.

### Fractional atomic coordinates and isotropic or equivalent isotropic displacement parameters (Å^2^)

**Table:**

	*x*	*y*	*z*	*U*_iso_*/*U*_eq_	
N1	0.4809 (3)	0.32620 (5)	0.62810 (11)	0.0311 (3)	
N2	0.3357 (3)	0.28455 (5)	0.63903 (13)	0.0379 (4)	
N3	0.4680 (3)	0.26168 (5)	0.73894 (13)	0.0395 (4)	
C4	0.7028 (3)	0.28997 (6)	0.79380 (14)	0.0321 (4)	
C5	0.9114 (4)	0.28229 (6)	0.90326 (15)	0.0374 (4)	
H5	0.9065	0.2539	0.9536	0.045*	
C6	1.1208 (4)	0.31732 (7)	0.93407 (15)	0.0411 (4)	
H6	1.2652	0.3131	1.0072	0.049*	
C7	1.1281 (4)	0.35962 (7)	0.86002 (16)	0.0412 (4)	
H7	1.2779	0.3831	0.8849	0.049*	
C8	0.9264 (3)	0.36815 (6)	0.75324 (15)	0.0348 (4)	
H8	0.9317	0.3967	0.7036	0.042*	
C9	0.7132 (3)	0.33208 (6)	0.72239 (13)	0.0292 (4)	
O10	0.3754 (2)	0.35998 (4)	0.53578 (9)	0.0327 (3)	
C11	0.4691 (4)	0.34745 (6)	0.42841 (14)	0.0328 (4)	
H11A	0.3904	0.3145	0.3939	0.039*	
H11B	0.6757	0.3455	0.4497	0.039*	
C12	0.3636 (3)	0.38958 (6)	0.33973 (13)	0.0286 (4)	
C13	0.5113 (3)	0.43544 (6)	0.35296 (13)	0.0296 (4)	
C14	0.4080 (3)	0.47548 (6)	0.27302 (14)	0.0316 (4)	
C15	0.1584 (3)	0.46843 (6)	0.18303 (14)	0.0335 (4)	
H15	0.0869	0.4957	0.1294	0.040*	
C16	0.0095 (3)	0.42330 (7)	0.16838 (13)	0.0318 (4)	
C17	0.1116 (3)	0.38296 (6)	0.24756 (13)	0.0299 (4)	
C18	0.7813 (3)	0.44365 (7)	0.45103 (15)	0.0389 (4)	
H18A	0.8466	0.4113	0.4906	0.058*	
H18B	0.7492	0.4676	0.5110	0.058*	
H18C	0.9231	0.4574	0.4151	0.058*	
C19	0.5633 (4)	0.52490 (7)	0.28165 (18)	0.0424 (4)	
H19A	0.7451	0.5190	0.2664	0.064*	
H19B	0.5917	0.5392	0.3628	0.064*	
H19C	0.4541	0.5487	0.2214	0.064*	
C20	−0.2599 (4)	0.41887 (8)	0.06843 (15)	0.0416 (4)	
H20A	−0.3033	0.4515	0.0268	0.062*	
H20B	−0.4120	0.4093	0.1031	0.062*	
H20C	−0.2393	0.3929	0.0106	0.062*	
C21	−0.0499 (4)	0.33394 (7)	0.23361 (17)	0.0399 (4)	
H21A	−0.0717	0.3235	0.3124	0.060*	
H21B	0.0518	0.3076	0.2029	0.060*	
H21C	−0.2348	0.3388	0.1765	0.060*	

### Atomic displacement parameters (Å^2^)

**Table:**

	*U*^11^	*U*^22^	*U*^33^	*U*^12^	*U*^13^	*U*^23^
N1	0.0347 (7)	0.0312 (7)	0.0230 (6)	−0.0011 (5)	0.0005 (5)	0.0022 (5)
N2	0.0426 (8)	0.0353 (8)	0.0313 (7)	−0.0056 (6)	0.0026 (6)	−0.0011 (6)
N3	0.0478 (9)	0.0343 (8)	0.0321 (7)	−0.0046 (6)	0.0038 (6)	0.0008 (6)
C4	0.0364 (8)	0.0301 (8)	0.0273 (8)	0.0026 (6)	0.0048 (6)	−0.0007 (6)
C5	0.0464 (10)	0.0351 (9)	0.0268 (8)	0.0078 (7)	0.0035 (7)	0.0042 (6)
C6	0.0385 (9)	0.0508 (10)	0.0275 (8)	0.0071 (8)	−0.0018 (7)	−0.0006 (7)
C7	0.0345 (9)	0.0477 (10)	0.0355 (9)	−0.0059 (7)	−0.0002 (7)	−0.0041 (7)
C8	0.0370 (9)	0.0347 (8)	0.0298 (8)	−0.0028 (7)	0.0044 (7)	0.0011 (6)
C9	0.0301 (8)	0.0318 (8)	0.0233 (7)	0.0041 (6)	0.0033 (6)	−0.0019 (6)
O10	0.0379 (6)	0.0353 (6)	0.0213 (5)	0.0086 (5)	0.0021 (4)	0.0026 (4)
C11	0.0380 (9)	0.0338 (8)	0.0248 (8)	0.0063 (7)	0.0055 (6)	−0.0015 (6)
C12	0.0305 (8)	0.0325 (8)	0.0222 (7)	0.0049 (6)	0.0062 (6)	−0.0013 (6)
C13	0.0281 (8)	0.0357 (8)	0.0240 (7)	0.0018 (6)	0.0057 (6)	−0.0040 (6)
C14	0.0339 (8)	0.0331 (8)	0.0295 (8)	0.0019 (6)	0.0114 (6)	−0.0019 (6)
C15	0.0349 (8)	0.0374 (9)	0.0275 (8)	0.0089 (7)	0.0077 (6)	0.0051 (6)
C16	0.0293 (8)	0.0428 (9)	0.0219 (7)	0.0046 (7)	0.0048 (6)	−0.0019 (6)
C17	0.0305 (8)	0.0351 (8)	0.0237 (7)	0.0011 (6)	0.0070 (6)	−0.0040 (6)
C18	0.0338 (9)	0.0460 (10)	0.0320 (9)	−0.0027 (7)	0.0009 (7)	−0.0053 (7)
C19	0.0456 (10)	0.0354 (9)	0.0483 (10)	−0.0028 (8)	0.0163 (8)	−0.0016 (8)
C20	0.0344 (9)	0.0580 (11)	0.0266 (8)	0.0056 (8)	−0.0012 (7)	−0.0019 (7)
C21	0.0380 (9)	0.0401 (9)	0.0403 (9)	−0.0046 (7)	0.0086 (7)	−0.0045 (7)

### Geometric parameters (Å, °)

**Table:**

N1—N2	1.3398 (19)	C13—C14	1.401 (2)
N1—C9	1.358 (2)	C13—C18	1.512 (2)
N1—O10	1.3741 (16)	C14—C15	1.392 (2)
N2—N3	1.304 (2)	C14—C19	1.505 (2)
N3—C4	1.383 (2)	C15—C16	1.388 (2)
C4—C9	1.390 (2)	C15—H15	0.9500
C4—C5	1.407 (2)	C16—C17	1.401 (2)
C5—C6	1.364 (3)	C16—C20	1.510 (2)
C5—H5	0.9500	C17—C21	1.508 (2)
C6—C7	1.410 (3)	C18—H18A	0.9800
C6—H6	0.9500	C18—H18B	0.9800
C7—C8	1.373 (2)	C18—H18C	0.9800
C7—H7	0.9500	C19—H19A	0.9800
C8—C9	1.396 (2)	C19—H19B	0.9800
C8—H8	0.9500	C19—H19C	0.9800
O10—C11	1.4714 (19)	C20—H20A	0.9800
C11—C12	1.501 (2)	C20—H20B	0.9800
C11—H11A	0.9900	C20—H20C	0.9800
C11—H11B	0.9900	C21—H21A	0.9800
C12—C13	1.402 (2)	C21—H21B	0.9800
C12—C17	1.412 (2)	C21—H21C	0.9800
			
N2—N1—C9	112.41 (13)	C15—C14—C13	118.49 (15)
N2—N1—O10	120.21 (12)	C15—C14—C19	120.09 (15)
C9—N1—O10	127.02 (13)	C13—C14—C19	121.41 (15)
N3—N2—N1	107.93 (13)	C16—C15—C14	122.80 (15)
N2—N3—C4	108.04 (14)	C16—C15—H15	118.6
N3—C4—C9	109.04 (14)	C14—C15—H15	118.6
N3—C4—C5	130.67 (16)	C15—C16—C17	119.15 (14)
C9—C4—C5	120.29 (15)	C15—C16—C20	119.39 (15)
C6—C5—C4	117.17 (16)	C17—C16—C20	121.45 (16)
C6—C5—H5	121.4	C16—C17—C12	118.77 (15)
C4—C5—H5	121.4	C16—C17—C21	119.69 (14)
C5—C6—C7	121.62 (16)	C12—C17—C21	121.53 (14)
C5—C6—H6	119.2	C13—C18—H18A	109.5
C7—C6—H6	119.2	C13—C18—H18B	109.5
C8—C7—C6	122.35 (16)	H18A—C18—H18B	109.5
C8—C7—H7	118.8	C13—C18—H18C	109.5
C6—C7—H7	118.8	H18A—C18—H18C	109.5
C7—C8—C9	115.62 (15)	H18B—C18—H18C	109.5
C7—C8—H8	122.2	C14—C19—H19A	109.5
C9—C8—H8	122.2	C14—C19—H19B	109.5
N1—C9—C4	102.58 (14)	H19A—C19—H19B	109.5
N1—C9—C8	134.47 (15)	C14—C19—H19C	109.5
C4—C9—C8	122.95 (15)	H19A—C19—H19C	109.5
N1—O10—C11	111.09 (11)	H19B—C19—H19C	109.5
O10—C11—C12	105.69 (12)	C16—C20—H20A	109.5
O10—C11—H11A	110.6	C16—C20—H20B	109.5
C12—C11—H11A	110.6	H20A—C20—H20B	109.5
O10—C11—H11B	110.6	C16—C20—H20C	109.5
C12—C11—H11B	110.6	H20A—C20—H20C	109.5
H11A—C11—H11B	108.7	H20B—C20—H20C	109.5
C13—C12—C17	121.24 (14)	C17—C21—H21A	109.5
C13—C12—C11	119.37 (14)	C17—C21—H21B	109.5
C17—C12—C11	119.33 (14)	H21A—C21—H21B	109.5
C14—C13—C12	119.53 (14)	C17—C21—H21C	109.5
C14—C13—C18	118.07 (15)	H21A—C21—H21C	109.5
C12—C13—C18	122.39 (14)	H21B—C21—H21C	109.5
			
C9—N1—N2—N3	−0.72 (19)	O10—C11—C12—C13	80.40 (17)
O10—N1—N2—N3	−174.26 (13)	O10—C11—C12—C17	−97.09 (16)
N1—N2—N3—C4	0.67 (18)	C17—C12—C13—C14	−0.3 (2)
N2—N3—C4—C9	−0.41 (19)	C11—C12—C13—C14	−177.69 (14)
N2—N3—C4—C5	179.01 (17)	C17—C12—C13—C18	179.83 (14)
N3—C4—C5—C6	179.99 (17)	C11—C12—C13—C18	2.4 (2)
C9—C4—C5—C6	−0.6 (2)	C12—C13—C14—C15	0.6 (2)
C4—C5—C6—C7	0.3 (3)	C18—C13—C14—C15	−179.49 (14)
C5—C6—C7—C8	0.1 (3)	C12—C13—C14—C19	−178.35 (14)
C6—C7—C8—C9	−0.1 (3)	C18—C13—C14—C19	1.6 (2)
N2—N1—C9—C4	0.44 (17)	C13—C14—C15—C16	−0.7 (2)
O10—N1—C9—C4	173.45 (14)	C19—C14—C15—C16	178.23 (15)
N2—N1—C9—C8	−179.69 (17)	C14—C15—C16—C17	0.5 (2)
O10—N1—C9—C8	−6.7 (3)	C14—C15—C16—C20	179.92 (15)
N3—C4—C9—N1	−0.01 (17)	C15—C16—C17—C12	−0.1 (2)
C5—C4—C9—N1	−179.51 (15)	C20—C16—C17—C12	−179.53 (14)
N3—C4—C9—C8	−179.90 (15)	C15—C16—C17—C21	179.26 (14)
C5—C4—C9—C8	0.6 (2)	C20—C16—C17—C21	−0.2 (2)
C7—C8—C9—N1	179.94 (17)	C13—C12—C17—C16	0.0 (2)
C7—C8—C9—C4	−0.2 (2)	C11—C12—C17—C16	177.45 (13)
N2—N1—O10—C11	−91.18 (17)	C13—C12—C17—C21	−179.36 (14)
C9—N1—O10—C11	96.30 (17)	C11—C12—C17—C21	−1.9 (2)
N1—O10—C11—C12	−174.71 (12)		

### Hydrogen-bond geometry (Å, °)

**Table:**

*D*—H···*A*	*D*—H	H···*A*	*D*···*A*	*D*—H···*A*
C18—H18C···Cg3^i^	0.98	2.85	3.6701 (18)	141
C20—H20B···Cg3^ii^	0.98	2.80	3.682 (2)	150

Symmetry codes: (i) *x*+1, *y*, *z*; (ii) *x*−1, *y*, *z*.
